# Nichtinvasive Bildgebung kutaner Melanommetastasen: Analyse von Mikromilieu und Gefäßstruktur unter Therapie

**DOI:** 10.1007/s00105-025-05550-9

**Published:** 2025-07-29

**Authors:** Oliver Mayer, Rebecca Schönherr, Julia Welzel, Sandra Schuh

**Affiliations:** Klinik für Dermatologie und Allergologie, Universitätsklinikum Augsburg, Sauerbruchstr. 6, 86179 Augsburg, Deutschland

**Keywords:** Malignes Melanom, Immuntherapie, Optische Kohärenztomographie, Line-field-konfokale optische Kohärenztomographie, Verlaufskontrolle, Malignant melanoma, Neoplasm metastasis, Immunotherapy, Optical coherence tomography, Noninvasive imaging

## Abstract

Ein 63-jähriger Patient mit kutanen Metastasen eines malignen Melanoms zeigte unter kombinierter Immuntherapie mit Ipilimumab und Nivolumab eine ausgeprägte therapeutische Antwort. Mithilfe von dynamischer optischer Kohärenztomographie (D-OCT) und Line-field-konfokaler optischer Kohärenztomographie (LC-OCT) konnte nichtinvasiv eine signifikante Reduktion der Gefäßdichte und -durchmesser beobachtet werden. Diese Veränderungen im Mikromilieu des Tumors korrelieren mit dem therapeutischen Effekt und unterstreichen das Potenzial der Bildgebung zur Verlaufskontrolle.

## Abstract (en)

A 63-year-old man with cutaneous melanoma metastases showed a strong therapeutic response to combined immunotherapy with ipilimumab and nivolumab. Using dynamic optical coherence tomography (D-OCT) and line-field confocal optical coherence tomography (LC-OCT), a significant reduction in vessel density and diameter was noninvasively observed. These vascular changes reflect the tumor microenvironment’s response to therapy and highlight the potential of noninvasive imaging in treatment monitoring.

## Anamnese und klinischer Erstbefund

**Abb. 1:**
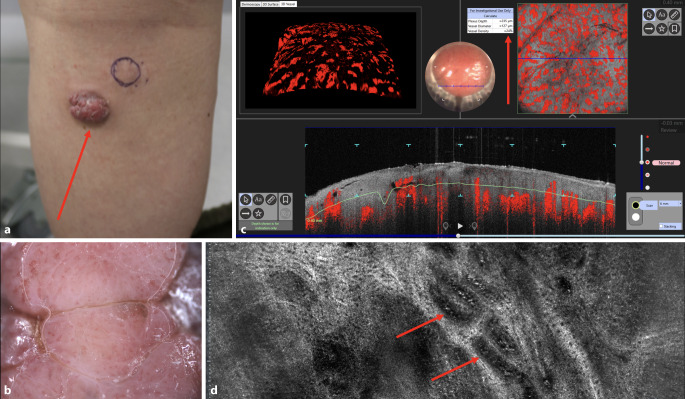
Metastase eines malignen Melanoms in der Kniekehle: **a** Makroskopisches Erscheinungsbild, aufgenommen mit der Kamera eines Apple iPhone 12 Pro (Apple Inc., Cupertino, CA, USA): exophytisch wachsender Knoten (*roter Pfeil*), subkutaner Knoten (*blauer Kreis*). **b** Dermatoskopisches Bild der exophytisch wachsenden Metastase: ausgeprägter vaskulärer Polymorphismus mit gepunkteten, geknäuelten, geschlängelten und Haarnadel-artigen Gefäßen (deepLive™, DAMAE Medical, Paris, Frankreich; dermatoskopisches Sichtfeld: 14 × 9 mm, Auflösung: 3,5 µm). **c** Dynamische optische Kohärenztomographie (D-OCT, Vivosight Dx, Michelson Diagnostics, Kent, Vereinigtes Königreich): großer Gefäßdurchmesser und hohe Gefäßdichte (*roter Pfeil*); diffuse und deutlich sichtbare Gefäße in horizontaler Ansicht (*oben rechts*, Bildgröße: 6 × 6 mm) und vertikaler Ansicht (*unten*, Bildgröße: 6 × 2 mm). **d** Line-field-konfokale optische Kohärenztomographie (LC-OCT, deepLive™ DAMAE Medical, Paris, Frankreich): dicke, geschlängelte Gefäße mit ausgeprägtem Blutfluss (*rote Pfeile*) (horizontale LC-OCT-Bildgröße: 1,2 × 0,5 mm)

Ein 63-jähriger Patient stellte sich im Februar 2024 mit einer seit etwa einem Jahr bestehenden exophytischen Hautveränderung am linken Unterschenkel vor (Abb. 1a, b). Zusätzlich bestanden 2 tastbare subkutane Knoten am linken Oberschenkel und in der Kniekehle. Allgemeinsymptome wurden verneint, Vorerkrankungen bestanden nicht.

Die Hautveränderung war erythematös, unregelmäßig begrenzt und leicht schuppend. Sie hatte sich über Monate hinweg langsam vergrößert.

## Histologie und molekulargenetische Analyse

Die histopathologische Untersuchung bestätigte ein malignes Melanom mit einer Tumordicke von 5,0 mm. Eine BRAF-V600-Mutation konnte nicht nachgewiesen werden. Dermatoskopisch zeigten sich multiple vaskuläre Strukturen mit typischem polymorphem Muster.

Histologisch handelte es sich um ein noduläres Melanom mit Ulzeration. Die Tumorzellen zeigten deutliche Pleomorphie und eine hohe Mitoserate. Immunhistochemisch war die Läsion positiv für S100 und HMB-45. Der Proliferationsindex (Ki-67) betrug etwa 60 %.

## Staging/Stadieneinteilung

Das Staging mittels diagnostischer Bildgebung (PET-CT [Positronenemissionstomographie-Computertomographie], cMRT [kraniale Magnetresonanztomographie]) ergab eine systemische Metastasierung mit kutanen Satelliten- und In-transit-Metastasen sowie den Befall retroperitonealer Lymphknoten und viszeraler Organe (Leber, Milz, Knochen) – entsprechend einem metastasierten malignen Melanom im Stadium IV (cM1c).

In der PET-CT zeigten sich multiple In-transit-Metastasen entlang der ipsilateralen Extremität, eine retroperitoneale Lymphadenopathie sowie ein hypermetaboler Fokus in Lebersegment VII. Die kraniale MRT ergab keinen Anhalt für zerebrale Metastasen.

## Immuntherapie mit Checkpointinhibitoren

Es erfolgte eine systemische Therapie mit Ipilimumab (3 mg/kgKG [Körpergewicht]) und Nivolumab (1 mg/kgKG) über 4 Zyklen. Die Behandlung wurde gut vertragen und führte zu einem makroskopisch sichtbaren Rückgang der kutanen Läsionen [1].

Die Kombination wurde leitliniengerecht im Rahmen einer metastasierten Erkrankung ohne BRAF-Mutation gewählt. Begleitend erfolgten engmaschige klinische und bildgebende Verlaufskontrollen [1].

## Nichtinvasive Bildgebung vor und nach Therapie

Zum Therapiemonitoring kamen dynamische optische Kohärenztomographie (D-OCT, Vivosight Dx, Michelson Diagnostics, Kent, Vereinigtes Königreich) und Line-field konfokale OCT (LC-OCT, deepLive™, DAMAE Medical, Paris, Frankreich) zum Einsatz.

Die Aufnahmen erfolgten prä- und posttherapeutisch an exakt derselben Hautstelle, identifiziert durch fotografische Dokumentation, anatomische Landmarken sowie teils gespeicherte Gerätekoordinaten. Die Gefäßparameter wurden mithilfe der Software des D‑OCT-Gerätes halbautomatisch analysiert, bei der LC-OCT erfolgte die Auswertung manuell. Als „region of interest“ diente jeweils bei der D‑OCT ein standardisierter Bildausschnitt (6 × 6 mm^2^).

Die angegebenen Werte sind arithmetische Mittelwerte aus 3 horizontalen Ebenen pro Zeitpunkt.

**Abb. 2:**
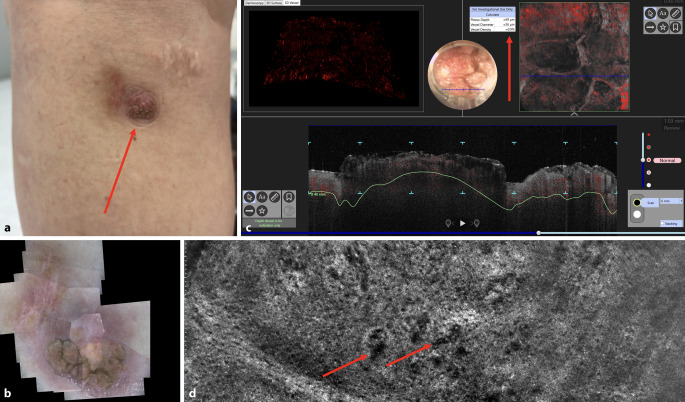
Dieselbe Metastase des malignen Melanoms wie in Abb. 1 nach 4 Zyklen Immuntherapie mit Ipilimumab und Nivolumab: **a** Makroskopische Ansicht, aufgenommen mit der Kamera eines Apple iPhone 12 Pro (Apple Inc., Cupertino, CA, USA): diskreter exophytischer Knoten (*roter Pfeil*). **b** Dermatoskopische Ansicht der Metastase: kaum sichtbare Gefäße (deepLive™, DAMAE Medical, Paris, Frankreich; dermatoskopisches Sichtfeld mit Mosaikfunktion: 14 × 9 mm, Auflösung: 3,5 µm). **c** Dynamische optische Kohärenztomographie (D-OCT, Vivosight Dx, Michelson Diagnostics, Kent, Vereinigtes Königreich): kleiner Gefäßdurchmesser und geringe Gefäßdichte (*roter Pfeil*); in der horizontalen Ansicht (*oben rechts*, Bildgröße: 6 × 6 mm) sind nur vereinzelt Gefäße (*Punkte, Knäuel, Linien*) sichtbar, in der vertikalen Ansicht (*unten*, Bildgröße: 6 × 2 mm) zeigt sich eine reduzierte Vaskularisation. **d** Line-field-konfokale optische Kohärenztomographie (LC-OCT, deepLive™, DAMAE Medical, Paris, Frankreich): dünne, diffuse Gefäße mit kaum erkennbaren Strukturen und minimal messbarem Blutfluss (*rote Pfeile*) (horizontale LC-OCT-Bildgröße: 1,2 × 0,5 mm)

Vor Therapie zeigten sich eine hohe Gefäßdichte (24 %) und ein mittlerer Gefäßdurchmesser von 127 µm (Abb. 1c). Nach der Immuntherapie waren diese Parameter deutlich reduziert: Dichte 0,9 %, Durchmesser 26 µm (Abb. 2c). Diese Veränderungen waren auch makroskopisch und dermatoskopisch sichtbar (Abb. 1a vs. 2a; Abb. 1b vs. 2b).

Die standardisierten Bildaufnahmen erfolgten unter reproduzierbaren Bedingungen in neutraler Lagerung. Die horizontale D‑OCT-Darstellung erlaubte die Analyse oberflächennaher Gefäßnetze, während die vertikale Aufnahme Aussagen zur Tiefe und Verteilung der Gefäße ermöglichte.

## Verlauf unter Therapie: klinisch und bildgebend

Die Läsionen zeigten nach 4 Zyklen eine deutliche Rückbildung. Makroskopisch blieb ein kleiner exophytischer Restknoten (Abb. 2a). Dermatoskopisch waren kaum noch vaskuläre Strukturen erkennbar (Abb. 2b). D‑OCT und LC-OCT belegten eine signifikante Reduktion der Gefäßarchitektur (Abb. 2c, d).

Die Bildgebung zeigte mikrovaskuläre Veränderungen bereits zu einem Zeitpunkt, an dem klinisch nur eine partielle Rückbildung sichtbar war. Dies deutet auf ein mögliches Frühansprechen hin, das durch herkömmliche makroskopische Beurteilung nicht erfasst wird.

In der LC-OCT-Analyse zeigten sich nach Therapie nur noch dünne, unregelmäßige Gefäße mit minimalem Blutfluss, was auf eine reduzierte Tumorvaskularisation hinweist (Abb. 2d). Die Bildgebung lieferte damit zusätzliche Hinweise zur immunvermittelten Tumorreaktion.

## Diskussion: Rolle der optische Kohärenztomographie-Verfahren im Monitoring

Diese Kasuistik zeigt exemplarisch, wie nichtinvasive Bildgebungsverfahren bei Patienten mit kutanen Metastasen eines malignen Melanoms zur Therapiebeurteilung beitragen können. Die mittels D‑OCT und LC-OCT beobachteten Veränderungen der Gefäßarchitektur lassen auf eine Umstrukturierung des Tumormikromilieus unter Immuntherapie schließen [2].

Während makroskopisch bereits eine klinische Besserung sichtbar war, ermöglichten die Bildgebungsdaten eine deutlich detailliertere Einschätzung auf mikrostruktureller Ebene. Die Reduktion der Gefäßdichte und des -durchmessers könnte mit einem immunvermittelten Rückgang der Tumorvaskularisation einhergehen, wie er in präklinischen Modellen unter Checkpointinhibition beschrieben wurde. Eine direkte Korrelation zwischen diesen Bildgebungsbefunden und immunologischen Mechanismen ist jedoch bislang nicht abschließend belegt [3, 4].

Der Einsatz von D‑OCT und LC-OCT kann bei gut zugänglichen kutanen Läsionen als ergänzende Methode zur klassischen klinischen Beurteilung herangezogen werden. Die Verfahren erlauben eine frühzeitige Detektion von Veränderungen, die mit einem Therapieansprechen assoziiert sein könnten.

Die nichtinvasive Bildgebung bietet somit die Möglichkeit, individuelle Verläufe dynamisch zu verfolgen und potenziell frühzeitig zwischen Responder- und Non-Responder-Situationen zu differenzieren [5]. In dieser Kasuistik wurde keine On-treatment-Biopsie durchgeführt, da eine klinisch eindeutige Rückbildung vorlag. Dennoch wäre die zusätzliche histologische Korrelation wünschenswert, um die Aussagekraft der vaskulären Bildgebungsmarker weiter zu validieren.

Die Anwendung der vorgestellten Technologien ist aktuell noch limitiert durch eine eingeschränkte Verfügbarkeit, hohe Kosten sowie das Fehlen standardisierter Auswertealgorithmen. Perspektivisch könnten quantitative vaskuläre Parameter aus der OCT-Bildgebung jedoch Teil integrierter Therapiealgorithmen werden – etwa zur Unterstützung frühzeitiger Therapieanpassungen im Sinne einer personalisierten Onkologie [6].

## Fazit für die Praxis

- D‑OCT (dynamische optische Kohärenztomographie) und LC-OCT (Line-field-konfokale optische Kohärenztomographie) ermöglichen die hochauflösende Darstellung vaskulärer kutaner Tumorveränderungen, können frühe Therapieeffekte objektivieren und die klassische Beurteilung ergänzen.
- Die Aussagekraft der vorliegenden Kasuistik ist aufgrund der Einzelbeobachtung naturgemäß limitiert.
- Perspektivisch sind prospektive Studien erforderlich, um die beschriebenen Parameter systematisch zu validieren und deren prognostische Relevanz im Verlauf der Immuntherapie zu untersuchen.

## Data Availability

Die den Ergebnissen dieser Kasuistik zugrunde liegenden Daten sind auf begründete Anfrage bei der korrespondierenden Autorin bzw. dem korrespondierenden Autor erhältlich.
